# Involvement of the *cbb*_3_-Type Terminal Oxidase in Growth Competition of Bacteria, Biofilm Formation, and in Switching between Denitrification and Aerobic Respiration

**DOI:** 10.3390/microorganisms8081230

**Published:** 2020-08-12

**Authors:** Igor Kučera, Vojtěch Sedláček

**Affiliations:** Department of Biochemistry, Faculty of Science, Masaryk University, Kotlářská 2, 611 37 Brno, Czech Republic; 21931@mail.muni.cz

**Keywords:** respiratory chain, terminal oxidases, denitrification, branched electron flow, biofilm

## Abstract

*Paracoccus denitrificans* has a branched electron transport chain with three terminal oxidases transferring electrons to molecular oxygen, namely *aa*_3_-type and *cbb*_3_-type cytochrome *c* oxidases and *ba*_3_-type ubiquinol oxidase. In the present study, we focused on strains expressing only one of these enzymes. The competition experiments showed that possession of *cbb*_3_-type oxidase confers significant fitness advantage during oxygen-limited growth and supports the biofilm lifestyle. The *aa*_3_-type oxidase was shown to allow rapid aerobic growth at a high oxygen supply. Activity of the denitrification pathway that had been expressed in cells grown anaerobically with nitrate was fully inhibitable by oxygen only in wild-type and *cbb*_3_ strains, while in strains *aa*_3_ and *ba*_3_ dinitrogen production from nitrate and oxygen consumption occurred simultaneously. Together, the results highlight the importance of the *cbb*_3_-type oxidase for the denitrification phenotype and suggest a way of obtaining novel bacterial strains capable of aerobic denitrification.

## 1. Introduction

Denitrifying organisms are, with possible rare exceptions, facultative anaerobes that constitutively use oxygen as the ultimate electron acceptor of their respiratory chain [1,2]. Under low oxygen tensions and in the presence of nitrate the core electron-transfer system is supplemented by the oxidoreductases of denitrification allowing for conversion of nitrate to dinitrogen coupled to the production of biological energy. Because of a number of points of electrons exit, the respiratory chains of denitrifiers are highly branched and their terminal branches compete with one another for electron flow coming from respiratory dehydrogenases. This gives rise to an “inhibition via respiratory chain” phenomenon whereby the reduction of one electron acceptor (e.g., nitrite or nitrous oxide) exerts a retarding effect on the reduction of another (e.g., nitrate) [3,4]. Oxygen usually strongly inhibits denitrification by the branch competition mechanism, although there are reported cases of bacterial strains able to denitrify under aerobic conditions [5,6]. The physiological basis for the lower oxygen sensitivity of aerobic denitrifiers is not clearly understood at the present time.

*Paracoccus denitrificans*, a common soil bacterium, is often studied as a model system for denitrification. Besides four enzymes of the denitrification pathway, namely the reductases of nitrate (Nar), nitrite (Nir), nitric oxide (Nor) and nitrous oxide (Nos), the bacterium produces three terminal oxidases of the heme–copper superfamily (reviewed in [7,8]). The presence of a *c*-type cytochrome-dependent oxidase activity is manifested by the cells’ ability to catalyze oxidation of N,N,N′,N′-tetramethyl-p-phenylenediamine (TMPD) to the Wurster’s blue radical cation and oxidative coupling of 1-naphthol and N,N-dimethyl-p-phenylendiamine to a blue indoaniline dye (the so-called “Nadi reaction”). In our early study we observed that the cyanide inhibition curve of TMPD oxidase was biphasic and the proportion of the more resistant component changed inversely with oxygen concentration in the inlet gas entering the culture chamber [9]. This result gave an indication that at low aeration the mitochondrial type (*aa*_3_) oxidase is complemented by another enzyme, later identified as a *cbb*_3_-type cytochrome *c* oxidase [10]. At the same time, a quinol oxidase was purified from the cytoplasmic membrane of *P. denitrificans* and shown to be a cytochrome *ba*_3_ [11]. Of the above three oxidase types, the *cbb*_3_-type is generally thought to have the highest affinity for dioxygen [12], although the real experimental values of *K*_M_(O_2_) significantly depend on the measuring technique, as has recently been demonstrated for *Pseudomonas aeruginosa* enzymes [13]. Given that oxygen fluctuations and limitation are common in soils [14], the possession of a *cbb*_3_-enzyme is likely to endow the bacterium with adaptive and competitive advantage over other microorganisms. There is metagenomic evidence for the widespread occurrence of the high-affinity terminal oxidase genes and hence bacteria with the potential to respire under microoxic conditions are expected to be abundant in nature [15].

The present paper deals with two linked issues. Firstly, we examined cell growth properties of “single route” *P. denitrificans* mutants that exclusively use only one of the terminal oxidases, *aa*_3_-, *cbb*_3_, or *ba*_3_-type. Specifically, we wanted to find out and quantify how the presence of a particular oxidase influences the growth in a mixed culture under conditions of limited and efficient aeration. The second research question posed concerned the capability of individual oxidases present in anaerobically grown mutant cells to mediate inhibition of denitrification activities by oxygen. Our expectation was that genetic mutations affecting terminal oxidases could improve the denitrification performance in an aerobic environment. The results provide evidence of a relationship between the inhibition of denitrification by oxygen and the presence of a *cbb*_3_-type oxidase.

## 2. Materials and Methods

### 2.1. Bacterial Strains

The *P. denitrificans* strains used in this study were kindly provided by Rob van Spanning (Vrije Universiteit Amsterdam, The Netherlands). They include: Pd1222 (parental strain, Rif^r^ [16]), Pd9311 (cytochrome *cbb*_3_ single-route mutant, Δ*cta*DI, Δ*cta*DII, *qox*B::Km^r^ [10]), Pd9312 (cytochrome *ba*_3_ single-route mutant, ΔctaDI, ΔctaDII, ccoNO::Km^r^ [17]), and Pd9233 (cytochrome *aa*_3_ single-route mutant, Δ*cco*NO, *qox*B::Km^r^ [18]).

### 2.2. Media

Cultures from glycerol stocks were first grown overnight in brain heart infusion (BHI) medium supplemented with appropriate antibiotics (rifampicin at 40 µg mL^−1^ for all strains and kanamycin at 25 µg mL^−1^ for the terminal oxidase mutant strains). For inocula and batch cultures, mineral media with succinate as sole carbon and energy source were used. The medium for aerobic growth contained 17 mM Na_2_HPO_4_, 33 mM KH_2_PO_4_, 50 mM NH_4_Cl, 1 mM MgSO_4_, 30 µM ferric citrate and 50 mM sodium succinate and was adjusted to a pH of 7.3. Anaerobic growth occurred in medium containing, in addition, 11 mM KNO_3_ and 0.6 mM Na_2_MoO_4_. Antibiotics were omitted from the final experimental cultures. 

### 2.3. Growth Competition Assays

Initial inocula were prepared by transferring 150 μL of BHI cultures to 100 mL Erlenmeyer flasks containing 15 mL of the mineral salt succinate medium and continuously agitating the flasks for 24 h at 250 rpm and 30 °C on a closed orbital shaker KS15A (Edmund Bühler GmbH, Bodelshausen, Germany). The resulting cultures were used to inoculate 30 mL of the same fresh medium at a final optical density at 600 nm (OD_600_) of 0.1, as measured in 1 cm cuvettes with a Ultrospec 2000 spectrophotometer (Pharmacia Biotech, Uppsala, Sweden). In case of mixed growth, the starting cultures were introduced at an OD_600_ ratio of 0.05:0.05. To achieve oxygen limitation during culturing, the shaking frequency was set at a minimum of 30 rpm. At zero time and after 48 h of growth, 100 μL of cultures were serially diluted to 10^5^, plated on agar and the plates were incubated at 30 °C for about four days. After the appearance of the colonies, the plates were flooded with a 1:1 mixture (*v/v*) of 1% 1-naphtol in 95% ethanol and 1% N,N-dimethyl-p-phenylenediamine monohydrochloride in water [19], drained, exposed to air for 10 min and the numbers of blue (Nadi-positive) and white (Nadi-negative) colonies were counted separately to obtain the values of colony-forming units (CFU) mL^−1^. The parameter W, which expresses the relative (Darwinian) fitness of the focal strain in comparison to the reference strain [20], was estimated as the ratio m_focal_/m_reference_, where m = log(CFU mL^−1^ at 48 h/CFU mL^−1^ at 0 h).

### 2.4. Aerobic Growth Study

Growth curves were generated in 200 μL cultures in succinate medium with initial OD_600_ of 0.1 in 96-well microplate format using an ELx808 microplate reader (BioTek Instruments Inc., Winooski, VT, USA). The plate was incubated at 30 °C, shaken continuously at 250 rpm and OD_600_ was automatically read at every 30 min for 20 consecutive h. OD_600_ values were averaged across 24 replicate cultures from the same inoculum. The maximum specific growth rate (µ_max_) was estimated as the slope of the tangent at inflection point of the average growth curve by applying the Gompertz-type model of Wijtzes et al. [21]. Data for biological replicates from two independent inocula were averaged. The difference in the two values of µ_max_ did not exceed 5%.

### 2.5. Biofilm Formation and Quantification

The ability of *P. denitrificans* strains to form a biofilm was measured by a Petri dish adherence assay with crystal violet staining as described by Kumar and Spiro [22]. Polystyrene Petri dishes of 6 cm diameter by 1 cm height were used each containing 10 mL of succinate mineral medium amended with 10 mM CaCl_2_ and inoculated by 130 μL of an overnight culture. After 72 h growth at 30 °C, the medium with planktonic cells was removed and its OD_600_ measured. The dishes were then stained with 0.1% crystal violet, washed and the amount of the dye bound to the biofilm was quantified by solubilizing in 20 mL of ethanol and measuring absorbance of the extracts at 595 nm. Results obtained from five dishes for each strain were averaged and presented relative to the wild-type level (100%).

### 2.6. Membrane Inlet Mass Spectrometry (MIMS) Measurements

Changes in the concentration of dissolved O_2_, N_2_ and N_2_O were monitored by a quadrupole mass spectrometer HPR-40 (Hiden Analytical, Warrington, UK) coupled with a dissolved species membrane probe provided by the manufacturer. The probe was inserted in the top of a 5-mL closed vessel, magnetically stirred and kept at 30 °C. The incubation medium (0.1 M sodium phosphate, pH 7.3, with 5 mM sodium succinate) contained initially 0.24 mM O_2_ and either 1 mM ^15^N-NaNO_3_ (98 atom%, Sigma-Aldrich, Prague, CR) or 0.24 mM ^14^N-N_2_O. Experiments were begun by the addition of 100 µL of cell suspension (5 mg dry weight). The detector (single channel electron multiplier) was set to monitor mass to charge (*m/z*) ratios of 32 for O_2_, 30 for ^15^N-N_2_, and 30 for ^14^N-N_2_O [23]. The membrane inlet mass spectrometry (MIMS) output was calibrated against standard solutions of known concentrations of gases. Based on the published reference data [24], the solubilities of N_2_, O_2_, and N_2_O in water at normal pressure were taken to be 0.62 mM (30 °C), 1.18 mM (30 °C), and 57.6 mM (0 °C), respectively.

### 2.7. Cytochromes c Spectrophotometry

The oxidation-reduction state of endogenous cytochromes *c* was monitored at 30 °C in a rubber-stoppered 1-cm cuvette with a Shimadzu UV-3000 dual-wavelength spectrophotometer as the absorbance difference between 550 and 535 nm. Washed suspension of anaerobically grown cells was diluted to 2.9 mg dry weight mL^−1^ in 3.2 mL of air-saturated (0.24 mM O_2_) 0.1 M sodium phosphate buffer, pH 7.3, containing 5 mM sodium succinate and in some experiments 0.17 mM sodium nitrate. Full oxidation of cytochromes was achieved by opening the cuvette and adding a few grains of potassium ferricyanide.

### 2.8. Statistical Analysis

Statistical analysis was mostly performed with Excel 2010 (Microsoft). All results are expressed as means ± standard deviations (SDs). One-way analysis of variance (ANOVA) served to compare values among the 3 groups. One sample and paired *t*-tests were used for comparisons of two values. The normality of data distribution was checked by the Shapiro–Wilk test using an online calculator found at http://www.statskingdom.com/320ShapiroWilk.html. The homogeneity of variance was tested by Excel´s F-test. The values of µ_max_ and their standard errors were obtained by nonlinear least-squares growth curves fitting in Origin 6.0 (Microcal Inc., Northampton, MA, USA).

## 3. Results

### 3.1. Comparative Growth Experiments

**Figure 1:**
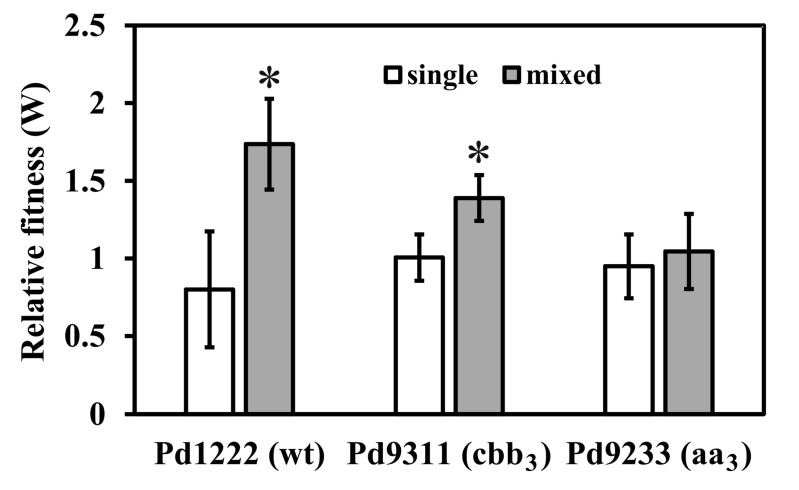
Relative fitness (W) of *P. denitrificans* strains versus the Pd9312 (*ba*_3_) strain as a reference. The results are from five separate experiments and represent means ± standard deviation (S.D.). Left bars, separate cultures; right bars, 1:1 mixed culture. The asterisk denotes statistically significant difference between the respective mixed culture and separate cultures (paired *t*-test, *p* < 0.01). There is no significant difference (analysis of variance (ANOVA), *p* = 0.48) in the W values for separate cultures (left bars) and none of these values differs significantly from 1 (one sample *t*-test, *p* > 0.05).

In order to simulate natural conditions where oxygen limitation often occurs, most cultivations were performed in flasks filled to 30% of the volume and minimally agitated. With this setup, the pattern of growth changed from exponential to linear when the cultures passed an OD_600_ of about unity after about 30 h. Cultivations were carried out for 48 h, after which the viable cell count expressed as CFU mL^−1^ was determined by agar plating and compared with the count at zero time. The Nadi-negative strain Pd9312, containing only the *ba*_3_-type oxidase, was used as a reference for determining relative growth rates of the remaining strains, which all are Nadi-positive. Cells of the tested strain and reference strain were grown either separately or in mixed culture and in both cases the relative fitness coefficient W was calculated as described under “Material and methods”. As Figure 1 shows, the strains grew at about the same rate when cultivated alone (W close to one). By contrast, in mixed cultures the outcome was clearly in favor of strains Pd1222 (wt) and Pd9311 (*cbb*_3_) (W equals to 1.74 and 1.39 respectively). The presence of the *cbb*_3_-type terminal oxidase thus provides the bacteria with a selective growth advantage when oxygen becomes scarce.

Growth properties of the strains were also examined by a microplate reader with continuous shaking. Based on the maximum specific growth rate, the four strains could be ranked in decreasing order as follows: Pd1222 (wt), 0.30 ± 0.01 h^−1^; Pd9233 (*aa*_3_), 0.280 ± 0.009 h^−1^; Pd9312 (*ba*_3_), 0.250 ± 0.004 h^−1^; Pd9311 (*cbb*_3_), 0.231 ± 0.006 h^−1^. These results thus suggest that the *aa*_3_-type oxidase is responsible for rapid growth under vigorous aeration.

### 3.2. Biofilm-Forming Ability

**Figure 2:**
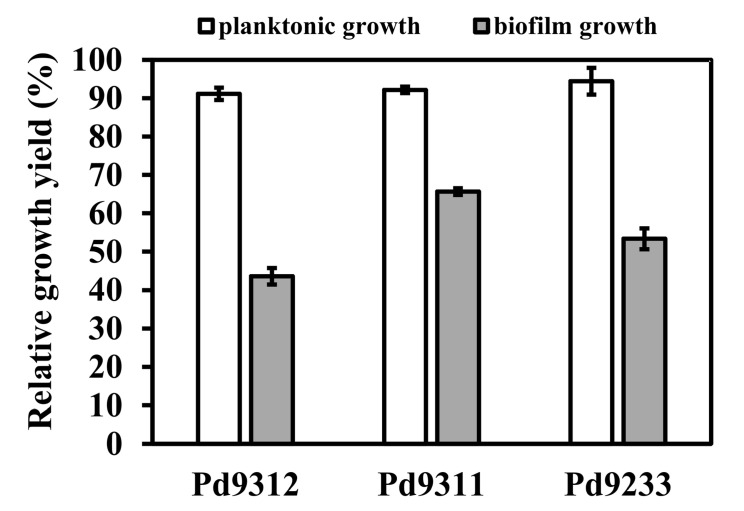
Planktonic bacterial growth (left bars) and biofilm formation (right bars) in polystyrene Petri dishes after 72 h. Data are normalized to wild-type values. Bar heights show mean values of five replicates, error bars show standard deviations. Means represented by left bars do not statistically differ (ANOVA, *p* = 0.12), while right bar values differ significantly from each other (*t*-test, *p* < 0.01).

Although non-motile, *P. denitrificans* can adhere to surfaces and create a biofilm in a calcium-dependent manner [22,25,26]. Therefore, we also assessed how the absence of some respiratory oxidases affected biofilm formation. Strains were grown for 24 h in succinate minimal medium supplemented with 10 mM CaCl_2_ in Petri dishes, and planktonic and biofilm biomass was quantified. The biofilm was found to be mainly developed on the walls of the dishes at the air–liquid interface. As shown in Figure 2, the concentration of planktonic cells did not vary much with the strains, but the amount of biofilm formed did. Among the mutant strains, Pd9311 showed the highest biofilm productivity, which amounted to approximately 66% of the wild-type level. This result speaks to a role of the high-affinity *cbb*_3_ oxidase in supporting the biofilm mode of growth.

### 3.3. Interaction between Aerobic Respiration and Denitrification

**Figure 3:**
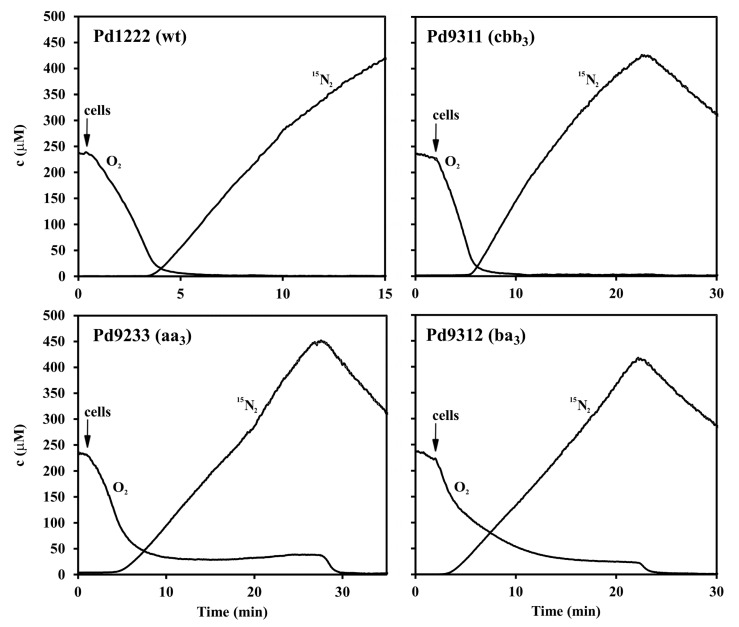
Dynamics of O_2_ consumption and production of N_2_ from nitrate in wild-type and single-oxidase strains of *P. denitrificans*. O_2_ and ^15^N_2_ concentrations were monitored by membrane inlet mass spectrometry (MIMS) at *m/z* 32 and 30 respectively. The measuring chamber was filled up with 5 mL of 0.1 M sodium phosphate pH 7.3, containing 5 mM sodium succinate, 1 mM ^15^N-NaNO_3_ and 0.24 mM O_2_. The arrow indicates addition of 100 μL of suspension of anaerobically grown cells (5 mg dry weight).

All the strains of *P. denitrificans* examined were capable of anaerobic growth with nitrate. The production of gas bubbles was apparent in all cases, demonstrating that denitrification of nitrate took place. To investigate the preference of each strain for electron acceptors, the harvested anaerobically-grown cells were injected into a closed chamber filled with a buffer containing succinate, oxygen and Na^15^NO_3_ and the change in concentration of O_2_ (*m/z* = 32) and ^15^N_2_ (*m/z* = 30) was monitored by membrane inlet mass spectrometry. The use of ^15^N isotope allowed to distinguish between the nitrogen originating from denitrification and the air-derived background nitrogen. From inspection of the time courses shown in the two upper panels of Figure 3, it is apparent that the strains Pd1222 and Pd9311 containing cytochrome oxidase *cbb*_3_ started denitrification only after the oxygen content had fallen to a low level. The strains Pd9233 and Pd9312 lacking this enzyme differed from the previous strains in their ability to reduce oxygen and nitrate simultaneously. From the lower two panels of Figure 3 it can be seen that in the presence of nitrate, the oxygen uptake rate gradually decreased in parallel with ^15^N_2_ accumulation and resumed again after nitrate consumption. In principle, the ^15^N_2_^+^ fragment of ^15^N_2_O might also contribute to the *m/z* 30 signal; parallel measurement at *m/z* 46, however, did not provide any evidence for elevated ^15^N_2_O accumulation. Therefore, we conclude that all four denitrification enzymes are aerobically active in strains Pd9233 and Pd9312.

**Figure 4:**
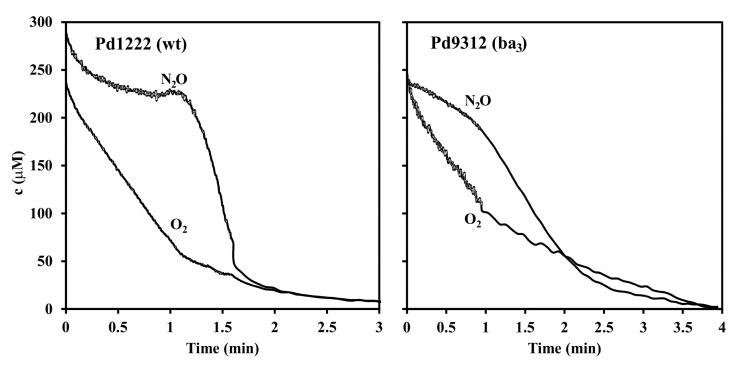
Kinetics of O_2_ and N_2_O respiration in wild-type and *ba*_3_ single-route strain of *P. denitrificans*. O_2_ and N_2_O concentrations were monitored by MIMS at *m/z* 32 and 30 respectively. The measuring chamber was filled up with 5 mL of 0.1 M sodium phosphate pH 7.3, containing 5 mM sodium succinate, 0.24 mM O_2_ and 0.24 mM N_2_O. The experiment was started by the addition of anaerobically grown cells (5 mg dry weight) at zero time.

The above conclusion was further corroborated for nitrous oxide reductase, the last enzyme of the denitrification pathway, by exposing the cells to a mixture of O_2_ and N_2_O. A typical experiment is shown in Figure 4. The co-respiration of both electron acceptors is evident from the simultaneous decrease in their concentrations (measured at *m/z* 32 and 30) that was seen for the Pd9312 mutant strain but not for the wild type strain. We also noted an increasing signal at *m/z* 28 as a sign of N_2_ accumulation. The interpretation of the *m/z* 28 signal, however, is not straightforward because significant parts of it arise from the N_2_^+^ fragment of the N_2_O added and from the CO^+^ fragment of the metabolically produced CO_2_.

**Figure 5:**
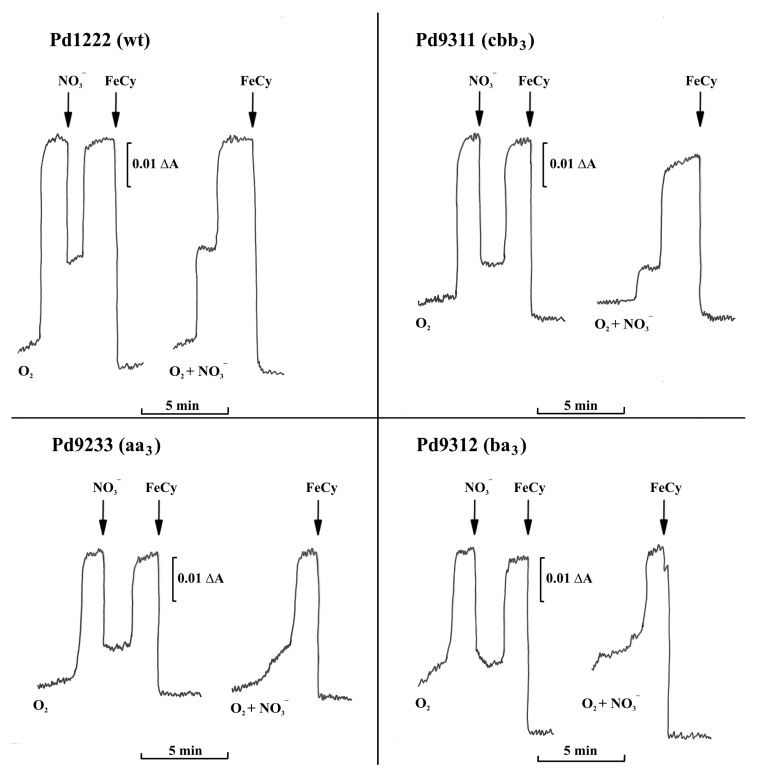
Electron acceptor-induced transient oxidation of cytochromes *c*. A closed 1-cm cuvette contained 3 mL of 5 mM succinate in 0.1 M sodium phosphate buffer (pH 7.3, 30 °C). At zero time, 9.3 mg dry weight of anaerobically grown cells were added and the time course of cytochrome *c* reduction was measured by dual wavelength spectroscopy at the wavelength pair 550 minus 535 nm. 0.24 mM O_2_ and/or 0.17 mM nitrate were present initially or at the times indicated by arrows. FeCy stands for potassium ferricyanide, added to attain the fully oxidized level.

The finding that the enzyme composition of the terminal part of the respiratory chain strongly influences partitioning of electron flow between O_2_ and NO_3_^−^ reduction prompted a subsequent evaluation of redox status of cytochromes *c*, electron donors for the key denitrification enzymes, by dual wavelength spectrophotometry (Figure 5). When a mixture of oxygen and nitrate was initially present, the reduction of cellular cytochromes *c* in strains Pd1222 and Pd9311 occurred with two distinct transient stages that were identical to those observed for the utilization of oxygen or nitrate added sequentially. A characteristic feature was that oxygen caused a significantly greater oxidation of cytochromes than did nitrate. This did not hold for strains Pd9233 and Pd9312 where the reduction states of cytochromes *c* in the presence of oxygen and nitrate were mutually comparable. The conclusion from the results in Figure 3, Figure 4 and Figure 5 is that the capacity of terminal oxidases of *aa*_3_-type and *ba*_3_-type is not sufficient to withdraw all electrons away from the denitrification enzymes.

## 4. Discussion

Possession by bacteria of multiple respiratory oxidases that vary in the affinity to oxygen is generally related to the ability to adapt to environments with fluctuating oxygen concentration. The present study provides a quantitative assessment of this idea through comparison of the relevant growth parameters for single oxidase strains derived from a three-oxidases-expressing parent strain. We have demonstrated that at a low aeration, the strains having the functional *cbb*_3_ oxidase can outcompete those that produce other types of oxidases (*aa*_3_ or *ba*_3_). On the other hand, this enzyme per se does not guarantee a high growth rate at high oxygen supply. Under such conditions, the mitochondrial type (*aa*_3_) oxidase becomes important. These findings are compatible with a view of the evolution of terminal oxidases, according to which the bacterial high affinity *cbb*_3_-type oxidases (C-type oxidases following the classification scheme of Sousa et al. [27]) originated from an NO-reducing ancestor soon after low initial levels of O_2_ had accumulated in the environment from photosynthesis, whereas the low affinity (A-type) oxidases could succeed in evolution only later when more oxygen was available [28,29].

With regard to biofilm formation, a comparison can be made with one of the most-studied biofilm formers, *Pseudomonas aeruginosa*. This bacterium produces highly structured biofilms with the thickness of up to several hundreds of micrometers that widely varies with the availability of oxygen [30]. The biofilms of an aerobically grown cytochrome *cbb*_3_ oxidases-deficient mutant strain were flatter and had less biomass compared with those of the parent strain, which indicated the necessity of microaerobic metabolism in oxygen-depleted zones [31]. Our data for *P. denitrificans* also support some role of the high-oxygen-affinity oxidase in biofilm development. The observed considerable interchangeability with other oxidases (Figure 2) can be explained by the fact *P. denitrificans* biofilms are only ∼4 μm thin [26] which is probably too low to significantly restrict oxygen diffusion, and therefore a high-affinity enzyme is not essential. The results also suggest that a single oxidase does not suffice for full biofilm development.

Similarly to many other denitrifying bacteria, *P. denitrificans* normally prefers oxygen over nitrogenous electron acceptors. In previous studies from our laboratory it has been shown that the natural preference can be weakened or even reversed by treatments diminishing electron flow to oxygen. A key role in promoting the observed aerobic-to-anaerobic respiratory transitions was ascribed to the denitrification intermediate nitric oxide that is hyper-produced under certain circumstances, freely diffuses in the solution and binds to terminal oxidases. Their inhibition results in a transient cessation of oxygen reduction and almost complete redirection of electron flow to the denitrification enzymes [32]. The present study further advances our knowledge of electron transport switching phenomena by showing that double deletion of the terminal oxidases per se can poise the respiratory chain electron carriers at a redox level allowing for functioning of denitrification pathway under aerobic conditions. This is especially so in the strain Pd9312 (*ba*_3_) in which oxygen cannot compete for electrons in the cytochromes *c* region because of the absence of the cytochrome *c*-linked terminal oxidases. 

Despite a number of reported isolates performing aerobic denitrification, no fundamental biochemical differences have been recognized so far between these strains and the classic denitrifiers. Our results indicate that these differences may be of a quantitative rather than qualitative nature, reflecting changes in the proportion between electron transfer capabilities of the reducing and oxidizing branches of the respiratory chain. Conthe et al. [33] related the preferential use of O_2_ over N_2_O observed in a natural mixed culture to the fact that the Monod half-saturation constant (*K*_s_) value for O_2_ was 1-2 orders of magnitude smaller compared to *K*_s_ (N_2_O). Although we found a clear association between deletion of the high-affinity oxidase and appearance of aerobic denitrification, the explanation based on the change in affinity for oxygen does not apply in our case because the effect of oxidase deletion persists even at high oxygen concentrations that saturate all terminal oxidases present. When considered in terms of Michaelis–Menten kinetic parameters of oxidases, a decrease in *V*_max_ is thus more effective than an increase in *K*_M_ (O_2_) in promoting aerobic denitrification. 

Aerobically denitrifying bacterial strains have biotechnological application potential in the removal of inorganic nitrogen compounds from contaminated wastewater, because reduction of nitrate and nitrite performed by them can take place simultaneously with aerobic oxidation of ammonium into nitrite and nitrate by nitrification. This simplifies the overall process and saves the costs [34,35]. The nitrous oxide reductase (Nos) reaction of the denitrification pathway is unique in that it represents the only biological sink of N_2_O, a greenhouse gas and a stratospheric ozone-depleting agent. Strategies employing Nos for controlling emissions of N_2_O are currently being developed, including transformation of plants with the *nosZ* gene for the Nos apoprotein or inoculation with genetically modified N_2_O-cracking strains [36]. In *P. denitrificans*, the *nosZ* (*pden_4219*) gene is upregulated at both the mRNA and the protein level in semiaerobically grown cells even in the absence of nitrogen electron acceptors [37,38]. Therefore, the *P. denitrificans* cultures may be useful for N_2_O bioremediation. Overall, our present findings can be considered as a step towards creation of new denitrifying strains capable of more effectively dealing with nitrogenous substances and functioning under a wide range of oxygen tensions.
